# The Prognostic Impact of HER2 Genetic and Protein Expression in Pancreatic Carcinoma—HER2 Protein and Gene in Pancreatic Cancer

**DOI:** 10.3390/diagnostics11040653

**Published:** 2021-04-04

**Authors:** Song-Hee Han, Ki Hyun Ryu, Ah-Young Kwon

**Affiliations:** 1 Department of Pathology, Dong-A University College of Medicine, Busan 49201, Korea; 2 Myunggok Medical Research Institute, Konyang University, Daejeon 35365, Korea; 3 Department of Internal Medicine, Division of Gastroenterology, Konyang University College of Medicine, Daejeon 35365, Korea; medidrug@kyuh.ac.kr; 4 Department of Pathology, CHA Bundang Medical Center, CHA University School of Medicine, Seongnam-si 13496, Korea; kwonahyoung@gmail.com

**Keywords:** pancreatic cancer, HER2, heterogeneity, prognosis

## Abstract

Pancreatic ductal adenocarcinoma (PDAC) is a lethal and clinically heterogeneous disease with a limited benefit from human epidermal growth factor receptor 2 (HER2)-targeted therapy. Recently, some studies have addressed the antitumoral effect of novel anti-HER2 drugs in HER2 low-expressing tumors. However, there have been few studies on the significance of low HER2 expression and genetic heterogeneity in PDAC. Using immunohistochemistry and dual-color silver-enhanced in situ hybridization based on the Trastuzumab for a gastric cancer scoring scheme, we evaluated HER2 protein expression, gene amplification, and genetic heterogeneity in three groups (HER2-neg, HER2-low, HER2-pos) of 55 patients. Among the 55 cases, 41.8% (23/55) showed HER2 expression of any intensity. HER2 amplification independent of HER2 expression was 25.5% (14/55). Patients in both these groups had a shorter overall survival than did patients in the HER2-neg group. HER2 genetic heterogeneity was identified in 37 (70.9%) of the 55 cases, mainly in HER2-neg and HER2-low groups. HER2 genetic heterogeneity significantly correlated with worse survival in the HER2-low and HER2-neg groups of PDAC. These findings support the hypothesis that low-level HER2 expression and heterogeneity have significant clinical implications in PDAC. HER2 heterogeneity might indicate the best strategies of combination therapies to prevent the development of subdominant clones with resistance potential.

## 1. Introduction

Pancreatic ductal adenocarcinoma (PDAC) is the fifth most common cause of cancer-related death in Korea [1]. PDAC has a 90% mortality rate, which is related to the difficulty of early detection, the general advanced stage at the time of diagnosis, and the limited availability of effective treatment options. Despite a massive research effort over the last few decades, the prognosis of PDAC has not improved significantly. Therefore, an understanding of the molecular characteristics of PDAC is urgently needed to select appropriate patients for the current treatment options available as well as the development of novel therapeutic targets.

The gene encoding HER2, known as ErbB-2, c-erbB2, or HER2/neu, is located on the long arm of chromosome 17 [2]. HER2 is normally expressed at low levels in a number of different tissues, such as epithelial, mesenchymal, and neuronal cells [3]. HER2 is functionally characterized by strong catalytic kinase activity. Activation of HER2 leads to increased signal transduction, resulting in cell proliferation, differentiation, and survival [4,5,6]. Amplification of HER2 on chromosome 17q21 and overexpression of the HER2 protein at the cell membrane have been associated with malignant transformation and poor survival rates in patients with breast cancer [7]. Moreover, numerous studies have shown that amplification of the gene and protein overexpression drive oncogenesis in different types of cancers, such as stomach, esophagogastric, ovary, and lung cancers [6,8,9]. Differential expression of HER2 at the gene and protein levels between normal and cancer cells and the prognostic significance of high HER2 levels support defining HER2 as an ideal selective target for patients with HER2-overexpressing cancer.

Trastuzumab is a recombinant humanized antibody that blocks the extracellular domain of the HER2 receptor tyrosine kinase present on cancer cells. Since the initial therapeutic effects of trastuzumab were published, it has been declared a revolution in cancer treatment [10]. This is because the drug confers outstanding disease-free and overall survival in patients with HER2-positive breast (BC) and gastric or gastroesophageal cancer (GC/GEC) [11,12]. However, in pancreatic cancer, HER2 overexpression has been reported in 0% to 82% of tumors, and its effects on prognosis are inconsistent [13,14,15]. Furthermore, several studies have not demonstrated improved survival of patients with HER2-positive PDAC.

Recently, a better understanding of tumor biology and HER2 signaling has led to the development and approval of new anti-HER2 therapeutic compounds. Anti-HER2 monoclonal antibodies consist of antibodies that bind with high affinity to different domains of HER2 [16,17]. Trastuzumab deruxtecan, a drug–antibody conjugate comprising the HER2 antibody trastuzumab with the topoisomerase I inhibitor deruxtecan payload via a synthetic linker, was approved in 2020 by the US Food and Drug administration for application in pretreated patients with HER2-positive BC who showed a poor response to other HER2-targeted therapies [18,19]. Preclinical studies have indicated that these therapies may also be effective against breast cancers with low HER2 expression, which is defined as a HER2 immunohistochemistry (IHC) score of 1 or 2 with a negative in situ hybridization (ISH) assay [18,19,20]. In addition, Gibbons-Fideler et al. [21] reported that BC cases with a HER2-negative IHC result and a positive fluorescence in situ hybridization result showed a good response to HER2-targeted therapy. Such a variation in the tumor treatment response paradoxically emphasizes the genomic heterogeneity of tumors as a result of the generation of a diverse cell population during tumor development and progression. Taken together, these findings might call into question previous research and reinforce the need to reconsider HER2 as a prognostic factor and to examine heterogeneity and the possible prognostic use of HER2 in PDAC.

In this study, we investigated HER2 status and the role of HER2 as a prognostic factor in terms of a therapeutic scenario to explore the opportunity of a more advanced treatment and the application of immunohistochemistry (IHC) and silver in situ hybridization (SISH). We categorized three groups of PDAC patients, HER2-positive, HER2-low, and HER2-negative, and analyzed the correlation between the presence of heterogeneity and prognosis.

## 2. Material and Methods

### 2.1. Study Design and Case Selection

We retrospectively enrolled patients with PDAC who were treated with radical surgery but without any preoperative therapy from January 2008 to December 2013 at Dong-A University Hospital and Konyang University Hospital. All cases were reviewed and staged by two pathologists according to the Eighth International Union against Cancer/American Joint Committee on Cancer (AJCC). Clinicopathologic data such as the patient’s age, sex, clinical stage, and pathological parameters were retrieved from medical records. Follow-up information including the patient’s survival and length of survival from the date of diagnosis until death were evaluated. Cases lost to follow-up were excluded from these analyses. A total of 55 patients with PDAC were enrolled in the present study. Seventeen of the patients with advanced cancer or presence of lymph node metastasis received post-operative adjuvant such as FOLFIRINOX (fluorouracil (5-FU), irinotecan, oxaliplatin), or Gemcitabine. Detailed data is shown in the Supplementary Materials Table S1. Additionally, 19 consecutive PDAC cases involving resection from January 2019 to December 2019 were assigned as a “validation set” for which the same enrolment criteria were applied. This study was approved by both Dong-A University Hospital and Konyang University Hospital Institutional Review Boards (DAUHIRB-18-234 & KYUH 2018-07-029).

### 2.2. Tissue Microarray Construction, HER2 Immunohistochemistry, and Silver-Enhanced In Situ Hybridization

Tissue microarray (TMA) methods were used to perform immunohistochemistry (IHC) and silver in situ hybridization (SISH). To reflect tumor heterogeneity, three 2-mm-diameter cores chosen from different representative areas of the carcinoma were reconstructed into a tissue array and arranged into new recipient blocks by SuperBioChips Laboratories (Seoul, Korea). One tissue core from invasive breast carcinoma with known HER2 gene amplification identified using SISH was included as a positive control. Negative control tissue cores from normal tonsils were also included. IHC was performed using 4-µm-thick formalin-fixed paraffin-embedded TMA sections and an automatic immuno-stainer (BenchMark ULTRA autostainer, Ventana, Tucson, AZ, USA). The primary antibody, anti-HER2 (4B5) (1:100 dilution; 10798; Ventana, Tucson, AZ, USA), was used according to the manufacturer’s instructions.

Assessment of HER2 amplification was performed using the dual-color SISH procedure. Briefly, unstained TMA slides were stained according to the protocol provided with the VENTANA BenchMark XT computerized automated slide stainer. The procedure combined two basic detection kits, HER2, and a chromosome 17 DNA probe cocktail (Ventana Medical System, Tucson, AZ, USA). The target gene (HER2) and corresponding Chromosome 17 (CEP 17) signals were detected as black and red signals, respectively.

### 2.3. Evaluation of HER2 Status Using HER2 Immunohistochemistry and Silver-Enhanced In Situ Hybridization

HER2 protein status was scored using the scheme for biopsy specimens of GC/GEC, as proposed by Hofmann et al. [22], used for the Trastuzumab for Gastric Cancer (ToGA) cohort of GC/GEC. The GC/GEC scoring system was applied when a small cluster of cells (≥5 neoplastic cells) showed a reaction and was performed as follows: negative (0), no reactivity, negative (1+), faint or barely perceptible membrane reactivity, equivocal (2+), weak to moderate complete or basolateral membrane reactivity, and positive (3+), strong complete or basolateral membrane reactivity. Cytoplasmic reactivity without membrane staining was defined as negative (0).

HER2 and CEP17 signals were enumerated in 50 cells after scanning each tissue core for areas with an increased number of HER2 signals using SISH. Gene amplification was defined as a HER2 to CEP17 signal ratio of ≥2.0. Cases with conflicting results of HER2 IHC and SISH were defined as discordant. Finally, we categorized the patients into three groups on the basis of HER2 protein and gene status: HER2-neg, HER2 IHC score of 0, HER2-low, HER2 IHC score of 1 or score 2 without gene amplification, and HER2-pos, HER2 IHC score of 3 and score 2 with gene amplification [20].

Intra-tumoral heterogeneity of the HER2 gene was evaluated according to College of American Pathologists (CAP) guidelines, as the existence of tumor cells with a HER2/CEP17 ratio >2.0 in 5–50% of the sum of tumor cells in the three cores. All pathologic examinations were evaluated independently by two experienced pathologists.

### 2.4. Statistical Analysis

Statistical analyses were performed using SPSS software (version 21.0; SPSS, Chicago, IL, USA). Chi-square and Fisher exact tests were applied to analyze associations between clinicopathologic variables and HER2 status and heterogeneity. We also used Spearman’s rank correlation coefficient to examine the relationship between HER2 protein and HER2 gene levels. To evaluate the prognostic value of HER2 in PDAC, overall survival (OS) was estimated using the Kaplan–Meier method and compared by the log-rank test.

## 3. Results

### 3.1. Analyses of the HER2 Protein and Encoding Gene Using IHC and SISH

The clinicopathological characteristics of both the main and validation cohorts are summarized in Table 1. The main cohort included 55 patients, with a mean patient age of 61.9 years (range, 31 to 81 years). The validation cohort included 19 patients, with a mean patient age of 67.2 years (range, 55 to 82 years).

Based on HER2 IHC staining of the main cohort, 32 cases (58.2%) scored 0, 14 cases (25.4%) scored 1+, 5 cases (9.1%) scored 2+, and 4 cases (7.3%) scored 3+. Twenty-three (41.8%) of the main cohort cases exhibited HER2 expression with any intensity according to IHC. HER2 amplification was detected in 14 cases (25.5%) (Table 2). Interestingly, of the 46 patients who were originally classified as negative for HER2 by IHC based on the GC/GEC IHC scoring scheme, nine were reclassified as having HER2 gene amplification (Table 2). Overall, there was a difference in the levels of HER2 gene and protein expression between the main sets and validation sets. However, it is more noteworthy that cases of HER2 IHC negativity with gene amplification were also identified in the validation set.

**Figure 1:**
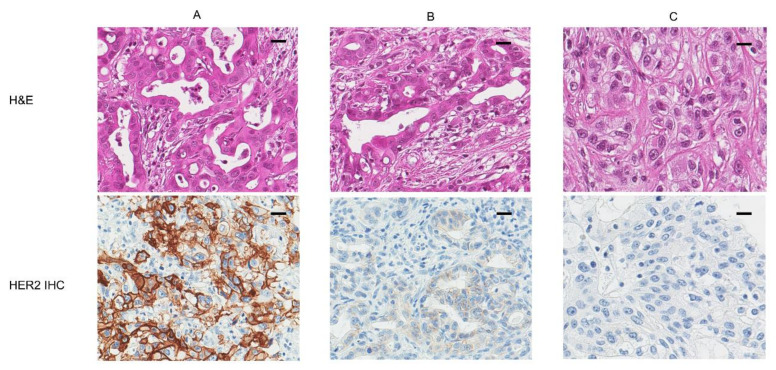
Representative examples of human epidermal growth factor receptor2 (HER2) protein. (**A**) A pancreatic adenocarcinoma showed overexpression of HER2 protein. (**B**) A pancreatic adenocarcinoma showed low expression of HER2 protein. (**C**) A pancreatic adenocarcinoma showed a negative expression (×200; scale bar, 50 µm).

In addition, we categorized 55 cases into three groups, as follows (Figure 1): 32 HER2-negative cases, 18 HER2-low cases, and 5 HER-positive cases. There was no statistically significant association between the classification of the three groups and clinicopathologic parameters.

### 3.2. Survival Analyses According to HER2 Protein and Gene Status in PDAC

**Figure 2:**
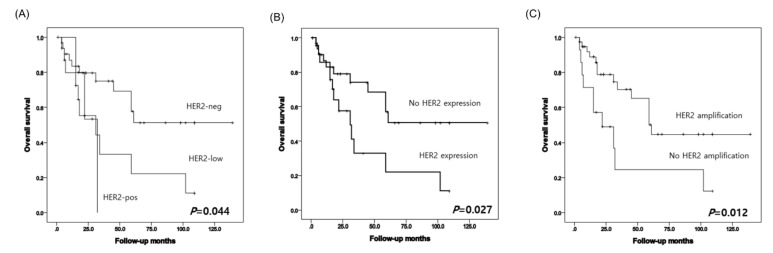
Kaplan-Meier survival analysis. The curve show overall survival differences according to HER 2 expression with three groups (**A**) and HER2 expression (**B**), and gene amplification status by HER2/CEP17 ratio (**C**).

When we analyzed the three groups of PDAC patients, HER2-neg, HER2-low, and HER2-pos, significant differences in survival were observed (*p* = 0.044) (Figure 2). Based on the HER2 scoring scheme, patients with HER2 IHC scores of 1, 2, and 3 had poorer overall survival (OS) than those with IHC scores of 0 (*p* = 0.027). Similarly, those with gene amplification based on the HER2/CEP17 ratio had a shorter OS than patients without amplification (*p* = 0.012). Nonetheless, discordance of IHC and SISH results was identified among patients with HER2 IHC scores of 0 and 1, and genetic heterogeneity was also identified. However, PDAC patients with HER2 overexpression did not show genetic heterogeneity.

### 3.3. Association of Clinicopathological Characteristics and HER2 Genetic Heterogeneity in HER2-Neg and HER2-Low Groups

The discordance of IHC and SISH results was 6.23% and 38.9% in the HER2-neg and HER2-low groups, respectively. HER2 genetic heterogeneity was identified in 37 (70.9%) of 55 PDAC cases, mainly in the HER2-neg and HER2-low groups. As shown in Table 3, HER2 genetic heterogeneity was associated with discordant HER2 IHC and SISH results. The heterogeneous group included cases with significantly higher HER2 IHC scores of 1 and 2 (*p* = 0.005) compared to the HER2 genetic non-heterogeneous group. In addition, positive HER2 amplification results were significantly related to the presence of genetic heterogeneity.

### 3.4. Survival Analyses in HER2-Neg and HER2-Low Groups

Overall, PDAC patients in the HER2-low and HER2-neg groups with HER2 genetic heterogeneity had significantly worse survival. However, this did not include all patients in the main cohort (*p* = 0.039). Moreover, we found that patients with HER2 amplification had reduced OS (*p* = 0.021)

## 4. Discussion

Assessment of HER2 status is key to therapeutic decision-making for patients with advanced carcinoma. Only HER2-positive cases defined by HER2 overexpression and HER2 amplification can be treated with anti-HER2 agents. Despite low HER2 expression, tumor cells with weak staining (1+) are considered negative for HER2 overexpression because such tumors are unlikely to respond to anti-HER2 therapy. New therapeutic compounds have been developed in recent years. In particular, antibody drug conjugates designed to target and deliver chemotherapy inside cancer cells have shown favorable risk benefits in patients with HER2-positive and HER2-low BC [18,19,20]. The novel HER2-targeting antibody–drug conjugate DS8201 inhibits the growth of both high- and low-HER2 expressing tumors [23]. In a previous study, Doi et al. [24] reported that DS8201 treatment has a significant advantage even in BC with low HER2 expression and in GC/GEC. The phase 1 study of ZW25, a HER2-targeted bispecific antibody, also showed promising antitumoral activity against tumors with low levels of HER2 expression [25]. Regardless, these promising data challenge the current concepts of HER2-targeted therapy in human cancers, including breast cancer and gastric cancer as well as pancreatic cancer. Nevertheless, previous studies on the prognostic significance of HER2 did not evaluate HER2 negativity or low-level HER2 expression. In fact, most investigators have grouped these categories together, assuming that a low expression level is not clinically meaningful. In the present study, we, for the first time, demonstrated that HER2 protein expression itself is associated with decreased survival, as is HER2 gene amplification, in PDAC. In particular, we identified the prognostic impact of low HER2 expression, as defined as an IHC score of 1 or 2, with SISH negativity. In addition, by evaluating the HER2 protein and gene status in PDAC, we confirmed that 18 patients (HER2-low, 32.8%) who were not eligible for trastuzumab might be eligible for new anti-HER2 therapeutic compounds. According to the research of Gibbons-Fideler et al. [21], nine patients (16.4%) with HER2 IHC negativity and HER2 gene amplification may benefit from emerging therapies. Notably, approximately 36.4% of patients with PDAC, including those with HER2 overexpression or HER2 equivocal expression with positive SISH results, might experience a therapeutic effect of anti-HER2 therapy.

Intra-tumoral heterogeneity (ITH) was first described in the 1830s by Muller [26], and with the rapid development of next-generation sequencing and mass spectrometry techniques, the importance of tumor heterogeneity is being fully realized. Even within the same tumor, significant heterogeneity from cell to cell can exist, resulting in substantial complexity in cancer [26]. In general, the prevalence of HER2 genetic heterogeneity in BC has been described in the range of 1–34% [27,28]. Other researchers have shown that intra-tumoral heterogeneity with regard to the HER2 gene amplification status is related to a significantly poorer prognosis than for those with homogeneous HER2 gene amplification in BC [29,30]. Although the organs involved differ, we identified the clinical significance of intra-tumoral heterogeneity of the HER2 gene in PDAC. Patients in the HER2-neg or HER2-low group showed higher genetic heterogeneity of HER2, and it was confirmed that genetic heterogeneity was associated with decreased survival.

The current treatment strategy of tumors is to attempt to target the principle alterations that are present in almost all cancer cells to eliminate the tumor. However, despite initial rapid and remarkable tumor regression, clinical drug resistance can develop later in the course of therapy, leading to therapeutic failure [31,32]. Arguably, heterogeneity is a central driver of therapeutic resistance and cancer recurrence, which is a critical issue in cancer treatment [33]. The concepts of evolutionary genetic changes in cancer and variant subclones that undergo stepwise alterations during tumor progression are unquestionable facts [34]. Tumor clones continue to mutate, and certain core molecular alterations might result in transcriptional differences, post-transcriptional modifications, or epigenetic dysregulation, which is different from the original progression [35,36]. In fact, studies have revealed that small populations of resistant cells may be present before treatment [37]. Thus, resistance may also occur as a result of the outgrowth of pre-existing treatment-resistant cells that suddenly find that they have acquired a survival advantage in the presence of a drug. In addition, some studies have demonstrated that the importance of subdominant clones within tumors lies in providing key paracrine signals to exert inter-clonal cooperation [38]. This clonal cooperation, together with the influence of the tumor stroma and the immune system, might cause the tumor to organize a functional association that is capable of altering all biochemical pathways required for tumor formation, synergistically contributing to tumor progression and metastasis [39].

There are some considerations of this study. First, the research in HER2 low settings combined with HER2-targeting antibody-drug conjugates is in the early stages and being trialed. Second, the increased level of HER2 protein may be beneficial from drug loading. However, what would need to be considered is that trastuzumab has several suppression mechanisms of HER2-dependent cell proliferation such as inhibiting HER2-receptor dimerization, preventing the sloughing of the extracellular domain, and immune activation [40]. Upon an immune reaction, some studies demonstrated that breast cancer cells with HER2 low levels resulted in initiating an immune response to breast cancer through the HER2 receptor, and the HER2 protein level was not the only factor indicating the drug response [41,42]. Nevertheless, a further study is required to clarify the mechanism of reaction in cancer with HER2 low expression.

HER2 is not the truncal alteration in PDAC. However, in our study, using IHC and HER2 amplification analyses showed that 41.8% and 25.5% of PDAC cases, respectively, exhibited HER2 expression with any intensity. We even demonstrated the presence of PDAC tumor cells with HER2 gene amplification in the HER2-neg group. As a subdominant clone in PDAC, targeting cells harboring HER2 may be an alternate or even complimentary approach from the perspective of eliminating the emergence of resistant cells to major drugs. Further studies are needed to confirm our observations with PDAC patients and investigate the opportunity of applying a new therapy approach for PDAC.

## Figures and Tables

**Table 1:** Characteristics of patients with pancreatic adenocarcinoma.

	Main Cohort	Validation Cohort
Characteristic	Number of patients (%)	Number of patients (%)
Cohort size	55 (100)	19 (100)
Sex		
Male	27 (49.1)	8 (42.1)
Female	28 (50.9)	11 (57.9)
Location		
Head	33 (60.0)	15 (78.9)
Tail	15 (27.3)	4 (21.1)
Body	6 (10.9)	0
Uncinate process	1 (1.8)	0
Tumor stage		
pT1	6 (10.9)	2 (10.5)
pT2	33 (60.0)	14 (737.7)
pT3	16 (29.1)	3 (15.8)
Node stage		
pN0	20 (35.7)	12 (63.2)
pN1	25 (44.6)	7 (36.8)
pN2	10 (17.9)	0
Clinical stage		
IA	4 (7.3)	1 (5.3)
IB	7 (12.7)	8 (42.1)
IIA	3 (5.5)	3 (15.8)
IIB	20 (36.3)	7 (36.8)
III	9 (16.4)	0
IV	12 (21.8)	0

**Table 2:** Comparison of HER2 immunohistochemistry (IHC) and Silver In Situ Hybridization (SISH) results.

HER2 Status	Main Cohort			HER2 Status	Validation Cohort		
IHC Score		SISH Result		IHC Score		SISH Result	
0	32 (58.2)	Ratio of ≥2.0	2 (6.3)	0	7 (36.8)	Ratio of ≥2.0	1 (14.2)
		Ratio of <2.0	30 (93.8)			Ratio of <2.0	6 (85.8)
1	14 (25.4)	Ratio of ≥2.0	7 (50.0)	1	5 (26.3)	Ratio of ≥2.0	1 (20.0)
		Ratio of <2.0	7 (50.0)			Ratio of <2.0	4 (80.0)
2	5 (9.1)	Ratio of ≥2.0	1 (20.0)	2	6 (31.6)	Ratio of ≥2.0	2 (33.3)
		Ratio of <2.0	4 (80.0)			Ratio of <2.0	4 (66.7)
3	4 (7.3)	Ratio of ≥2.0	4 (100.0)	3	1 (5.3)	Ratio of ≥2.0	1 (100.0)
Total (n, %)	55 (100)			Total (n, %)	19 (100)		

HER2, Human epidermal growth factor receptor 2. IHC, Immunohistochemistry. SISH, Dual-color silver-enhanced in situ hybridization.

**Table 3:** Clinicopathologic correlation of pancreatic adenocarcinoma with HER2-neg and HER2-low and intra-tumoral genetic heterogeneity.

Characteristics	HER2 Heterogeneity (n = 36)	HER2 Non-Heterogeneity (N = 14)	*p*
Age (year)	62.3 ± 8.6	59.8 ± 9.3	
Tumor size (cm)	3.6 ± 1.7	3.9 ± 1.9	
Tumor stage			0.129
pT1	2 (5.6)	3 (21.4)	
pT2	25 (69.4)	6 (42.9)	
pT3	9 (25.0)	5 (35.7)	
Node stage			0.753
pN0	15 (41.7)	5 (35.7)	
pN1-2	21 (58.3)	9 (64.3)	
HER2/CEP17 ratio	1.8 ± 0.55	1.7 ± 0.12	
HER2 gene amplification			0.039
Negative	27 (75.0)	14 (100.0)	
Positive	9 (25.0)	0 (0)	
HER2 IHC score			0.005
0	20 (55.6)	12 (85.7)	
1	12 (33.3)	2 (14.3)	
2	4 (11.1)	0 (0)	
Discordance of IHC and SISH			0.045
No	26 (72.2)	14 (100.0)	
Yes	10 (27.8)	0 (0)	

HER2, Human epidermal growth factor receptor 2. PDCA, Pancreatic adenocarcinoma. HER2/CEP17, ratio of HER2 genes to centromere 17.

## Data Availability

The data used to support the findings of this study are available from the corresponding author upon request.

## Supplementary Materials

Supplementary materials can be found at https://www.mdpi.com/article/10.3390/diagnostics11040653/s1. Table S1. Treatments and HER2 status of patients of pancreatic adenocarcinoma.
