# Supplementary material for: The Prognostic Impact of HER2 Genetic and Protein Expression in Pancreatic Carcinoma—HER2 Protein and Gene in Pancreatic Cancer
Source: Diagnostics (Basel). 2021 Apr 4;11(4):653. doi: 10.3390/diagnostics11040653 (PMC8065582; doi:10.3390/diagnostics11040653)
Supplement: Supplementary file 1 [file diagnostics-11-00653-s001.pdf]

Table S1. Treatment regarding HER2 status/

	Surgery + postoperative adjuvant therapy
HER2 status	
HER2-neg	9
HER2-low	7
HER2-pos	1
HER2 protein status	
No HER2 expression	9
HER2 expression	8
HER2 gene status	
No HER2 amplification	11
HER2 amplification	6
